# Sensory Deficit Following Anterior Cruciate Ligament Reconstruction with Bone–Patellar Tendon–Bone Autograft: Platelet-Rich Fibrin (PRF) Could Provide a Solution

**DOI:** 10.3390/medicina61122202

**Published:** 2025-12-12

**Authors:** Darko Milovanovic, Marko Kadija, Dusica Gavrilović, Svetlana Sreckovic, Miljan Bilanovic, Aleksandar Matić, Petar Vukman

**Affiliations:** 1 Clinic for Orthopedic Surgery and Traumatology, University Clinical Center of Serbia, Pasterova 2, 11000 Belgrade, Serbia; prvukman@gmail.com; 2 School of Medicine, University of Belgrade, Dr Subotica 8, 11000 Belgrade, Serbia; 3 Euromedik Hospital, Bulevar Umetnosti 29, 11000 Belgrade, Serbia; 4 Institute for Oncology and Radiology of Serbia, Pasterova 14, 11000 Belgrade, Serbia; 5 Center for Anesthesiology and Resuscitation, University Clinical Center of Serbia, Pasterova 2, 11000 Belgrade, Serbia; 6 University Hospital Medical Center Bezanijska Kosa, Dr Zorza Matea bb, 11000 Belgrade, Serbia; 7 Clinic for Orthopedics and Traumatology, University Clinical Centre of Kragujevac, Zmaj Jovina 30, 34000 Kragujevac, Serbia; 8 Faculty of Medical Sciences, University of Kragujevac, Svetozara Markovica 69, 34000 Kragujevac, Serbia

**Keywords:** ACL reconstruction, BPTB graft, sensory deficit, numbness, platelet-rich fibrin

## Abstract

*Background and Objectives*: Despite the high rate of donor site morbidity, a bone–patellar tendon–bone (BPTB) graft remains the gold standard when choosing a graft for anterior cruciate ligament (ACL) reconstruction. Damage to the infrapatellar branch of the saphenous nerve (IPBSN) during graft harvesting results in sensory deficits. Despite its high occurrence in the postoperative period, many patients go untreated, leading to a lower quality of life and potential professional impairment. The aim of this study was to assess the effectiveness of PRF therapy in alleviating sensory deficits and enhancing sensory nerve function in patients who have undergone BTB ACL reconstruction. *Materials and Methods*: This study was registered at ClinicalTrials.gov (Name of registry: ClinicalTrials.gov; Trial registration number: NCT07257666; Date of registration: 2 December 2025; Study start date: 7 January 2022). Over a one-year period, the pilot study enrolled 53 patients, treated them with BPTB ACL reconstruction, and divided them into two groups. The testing group’s donor site and subcutaneous tissue were treated with Vivostat^®^ PRF, whereas the standard group’s donor site and subcutaneous tissue remained untreated. The primary outcome measured was a reduction in the subjective numbness, which was tested during follow-up checks. Secondary outcomes included the evaluation of subjective knee scores for functional recovery, reported by the patients on control exams. *Results*: The use of Vivostat^®^ PRF resulted in a statistically significant reduction in sensory deficit among the groups at eight months (*p* < 0.05) and twelve months (*p* < 0.01) following surgery, favoring the testing group. The most substantial decrease in symptomatic patients was observed between four and eight months post-surgery, with no statistically significant difference found between the eight- and twelve-month follow-ups (*p* > 0.05). Evaluations of subjective knee function and activity scores showed no statistically significant differences between the groups. *Conclusions*: Using Vivostat^®^ PRF helps reduce sensory impairment in the area and minimizes donor site morbidity after BPTB ACL reconstruction.

## 1. Introduction

**Figure 1:**
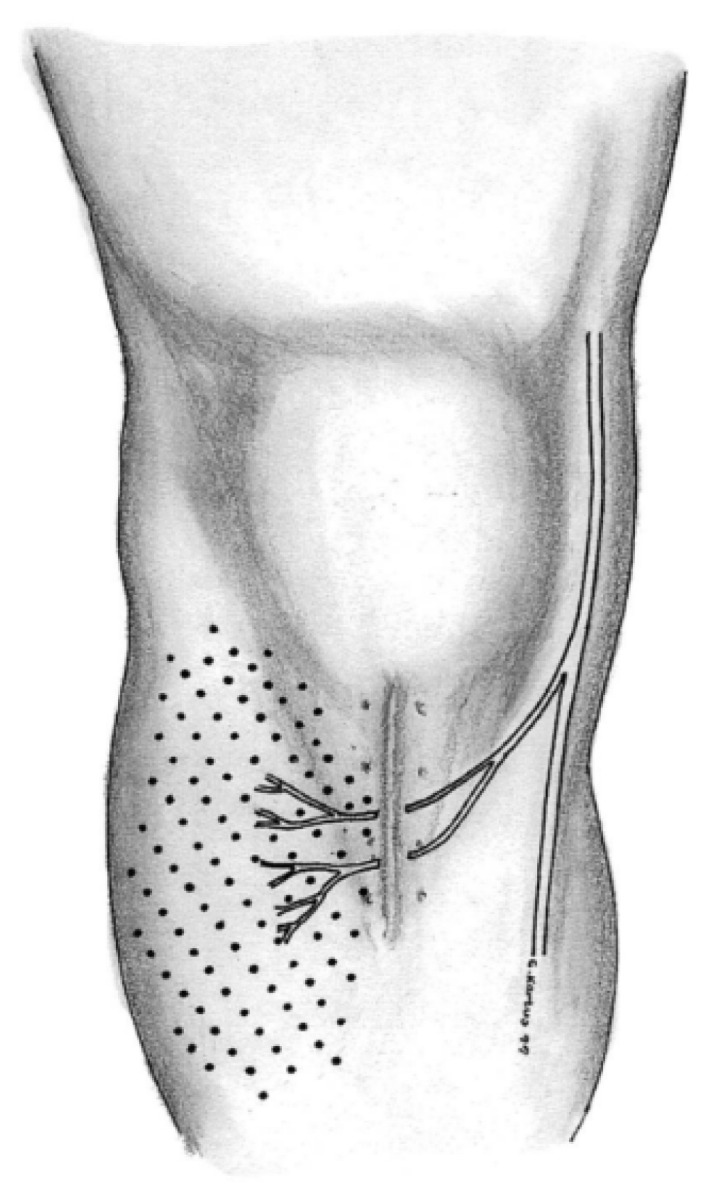
Surgical incision and lesion of the infrapatellar branch during graft harvesting and consecutive sensory deficit [10].

Anterior cruciate ligament (ACL) reconstruction is one of the most commonly performed surgical procedures in orthopedic surgery [1]. Despite the significant donor site morbidity associated with bone–patellar tendon–bone (BPTB) grafts, many surgeons still consider them the gold standard for graft selection [2]. Some studies suggest that the actual incidence of donor site morbidity is lower than reported in the literature [3]. BPTB graft harvesting can result in pain in the anterior knee segment, kneeling pain, and sensory deficits, with severe complications such as patellar fractures and ruptures of the patellar ligament also possible [4,5]. Sensory deficits may manifest as numbness, dysesthesia, or anesthesia, sometimes accompanied by painful neuromas and anterior knee pain [6,7]. Numbness occurs in up to 88% of patients following BPTB ACL reconstruction [8]. This deficit can persist for months or even years, and in some cases, it may become permanent. Though extremely rare, the occurrence of neurological deficits during arthroscopic meniscus or cartilage surgery has been described in the literature [9]. The cause of numbness lies in the neurological impairment of the infrapatellar branch of the saphenous nerve (IPBSN), which can occur either during surgical incision for graft harvesting or during the creation of portals for arthroscopic surgery (Figure 1).

**Figure 2:**
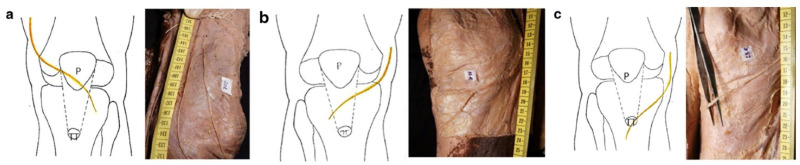
Anatomic variation in one IPBSN: (**a**) ending in line of the patellar(P); (**b**) ending in line of patellar ligament; (**c**) ending in line of tibial tubercle (TT) [13].

Anterior knee neuroanatomy is highly complex, requiring a thorough understanding to properly address associated issues and potentially prevent symptom development. The saphenous nerve is a sensory branch of the femoral nerve that innervates the skin over the patella, patellar ligament, medial lower leg, ankle and foot. At the knee level, it lies between the first and second layers, giving off branches that cross anterior to the patellar ligament, forming the infrapatellar plexus [11,12]. The occurrence and extent of sensory deficits depend on anatomical variations in this terminal branch. Studies have shown that, in most cases, there is only one infrapatellar branch, either superior or inferior, while in rare instances, two branches—superior and inferior—are present, with one typically predominant [6]. When only one branch is present, its course follows one of three anatomical patterns: over the patella, between the patellar apex and tibial tuberosity, or at the tibial tuberosity level (Figure 2) [13].

**Figure 3:**
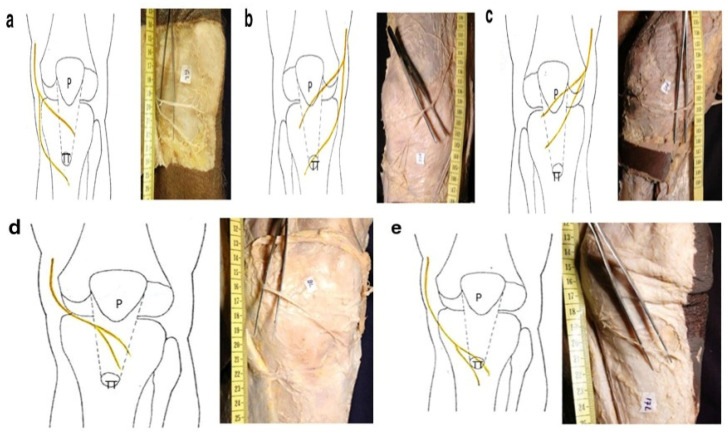
Anatomic variation in two IPBSN: (**a**) one branch at level of the patellar ligament, other at tibial tubercle (TT); (**b**) one branch at the level of patella (P) and other at tibial tubercle (TT); (**c**) one branch at patella and other of the patellar ligament; (**d**) both branches at the level of patellar ligament; (**e**) both branches at the level of tibial tubercle (TT) [13].

In rare cases where two terminal branches exist, their trajectories are significantly more complex. Anatomical studies show that the superior branch may be located at varying levels between the patellar apex and tibial tuberosity, while the inferior branch is typically distal to the tibial tuberosity. Another variation involves the superior branch at the patellar apex and the inferior branch at the tibial tuberosity. A third pattern positions the superior branch at the patellar apex and the distal branch between the patellar apex and tibial tuberosity. The fourth variation localizes both terminal branches at the patellar ligament, while the fifth is characterized by a substantially distal distribution of both branches at the tibial tuberosity (Figure 3) [13]. Further branching is described as variable distribution of the ramus, though some studies report consistent branching between the patellar apex and tibial tuberosity, and, less frequently, at the patellar apex or tibial tuberosity itself [6,13].

Bioregenerative medicine products are widely accepted in orthopedic practice, with platelet-rich plasma (PRP) currently being the most popular due to its broad clinical applications [14]. Their primary advantage lies in their regenerative potential, attributed to the numerous cytokines and growth factors they contain. Advancements in technology have led to the development of other products, including platelet-rich fibrin (PRF). The Vivostat^®^ System (Vivostat A/S, Lillerod, Denmark) enables the concentration of autologous fibrin matrix, producing a final product with up to seven times higher concentrations of platelets and fibrin, while simultaneously reducing matrix metalloproteinase-9 concentration, which is an enzyme that inhibits tissue healing [15]. Due to this composition, PRF has a great potential to induce and accelerate soft tissue regeneration [16,17]. A study conducted by Skarpas G. showed that the use of PRF enables a local effect lasting up to 12 months after application, leading to improved functional recovery [18]. Animal studies conducted on injured peripheral nerves in rats have demonstrated positive effects on functional nerve recovery following the use of platelet-rich plasma (PRP) and platelet-rich fibrin (PRF), although there was no improvement noted in the histomorphometric analysis [19]. Additionally, PRP has been shown to positively influence remyelination and functional recovery [19]. Other studies have indicated that PRP stimulates Schwann cell proliferation and increases neurotrophin levels, which play a crucial role in nerve healing after injury [20]. Research by Lambrichts et al. revealed promising results regarding leukocyte-platelet-rich fibrin in enhancing nerve regeneration and the innervation of damaged peripheral tissues [21]. While we identified only a few human studies involving PRF, one study showed promise. The research conducted by Kuffler et al. suggests that the use of PRF may facilitate sensory and motor recovery following peripheral nerve trauma [22].

Since this condition is relatively common and does not result in significant functional limitations postoperatively, many surgeons leave it untreated. However, given that sensory deficits can impair quality of life and cause difficulties in professional activities, they should not be overlooked; rather, the issue must be properly addressed. Current literature lacks data on the treatment of iatrogenic injury to the infrapatellar branch of the saphenous nerve (IPBSN) following anterior cruciate ligament reconstruction (ACLR), which could accelerate sensory recovery. In our study, we focused on the use of bioregenerative medicine products, such as platelet-rich fibrin (PRF), hypothesizing that they may enhance sensory recovery in patients undergoing ACLR with bone–patellar tendon–bone (BPTB) grafts.

## 2. Materials and Methods

### 2.1. Study Background

**Chart 1:**
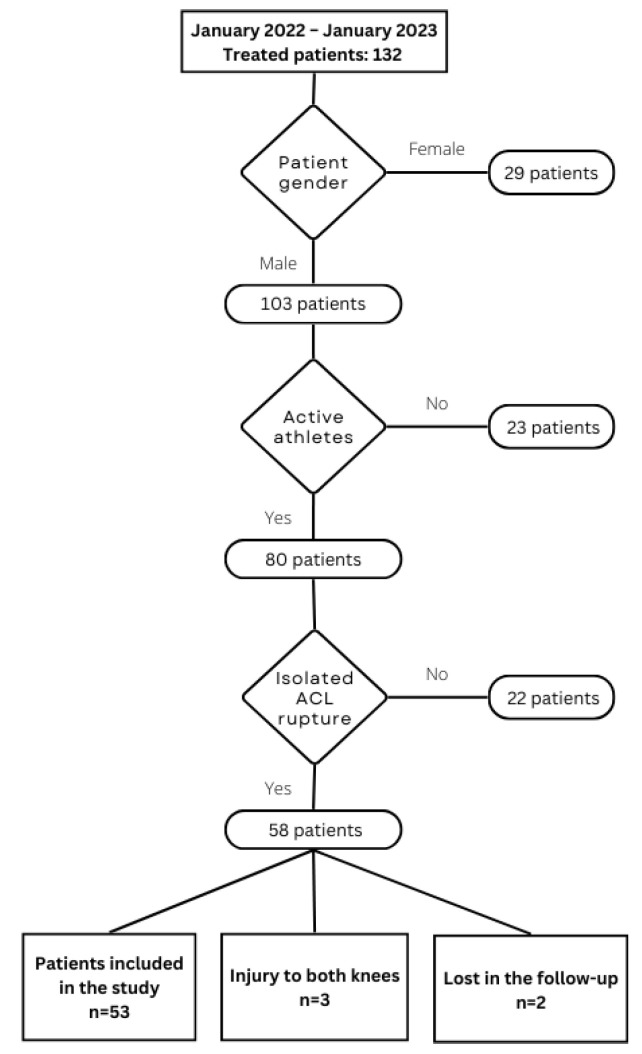
Patient selection process.

This study was initially designed and conducted as a single-center pilot cohort study at a time when prospective registration in ClinicalTrials.gov was not required by our institutional policies or local regulations for this type of investigation. As a result, the trial was registered retrospectively at ClinicalTrials.gov (NCT07257666; registration completed on 2 December 2025, after patient enrollment and primary data collection had been completed. The research was designed as a pilot cohort study and was conducted at the XXBLINDEDXX. It received approval from the Ethics Committee University Clinical Centre of Serbia under reference number 578/5, and Ethics Committee School of Medicine University of Belgrade under reference number 17/VI-3. Between January 2022 and January 2023, ACL reconstruction using a BPTB graft was performed on 53 patients who met all inclusion criteria by one orthopedic surgeon and an identical surgical team. The inclusion criteria were: male patients actively participating in sports, isolated ACL rupture, as well as intact sensation in the anterior knee. Exclusion criteria were: failure to attend follow-up appointments, previous surgery on the same or contralateral knee, or meniscal or cartilage injuries. All patients signed informed consent forms after being thoroughly briefed on the study’s objectives and potential effects. The patient selection process is illustrated in Chart 1. We formed two cohorts of patients: the Vivostat group (Group 1, donor site filled with autologous PRF-Vivostat A/S, Alleroed, Denmark, 24 patients) and the standard group (Group 2, donor site left unfilled, 29 patients).

Upon hospital admission and informed consent form signing, all patients completed the Tegner activity score to assess physical activity levels. Additionally, they completed subjective knee function assessment questionnaires for the four-week preoperative period, including: Modified Cincinnati score, International Knee Documentation Committee (IKDC) score and Tegner–Lysholm score.

### 2.2. Vivostat^®^ Preparation and Surgical Technique

Immediately before anesthesia induction, 120 mL of venous blood was collected from all patients in the experimental group for platelet-rich fibrin (PRF) preparation. The PRF processing took place in the operating room during surgery, without the need to further prolong the surgery time. The preparation was conducted using the Vivostat^®^ Processor Unit according to the manufacturer’s recommendations and lasted approximately 25 min, resulting in 5–6 mL of the final PRF product, which was then loaded into the Applicator Unit and applied to the surgical site using a spray pen. We applied the product using a low setting on the application unit, with the spray pan positioned 10 cm from the surgical site. With this configuration, the delivery rate was 0.7 mL/min, and the average delivery time was 85 s, with an air force of 4.9 mN directed at the target site, as indicated by the manufacturer’s data [23].

**Figure 4:**
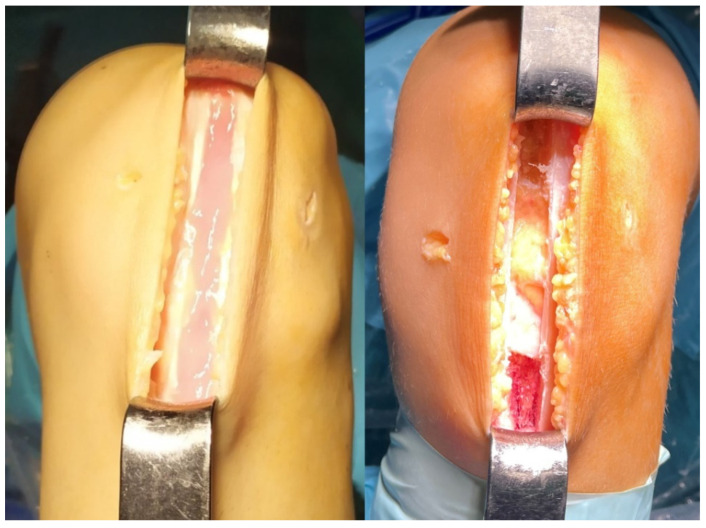
Defect filled with Vivostat^®^ in the experimental group (**left**); Defect left unfilled in standard group (**right**).

The surgical technique followed an anatomic reconstruction approach. The graft was harvested through a longitudinal incision, approximately 10 cm in length, positioned along the medial border of the patellar ligament. The paratenon was carefully identified and meticulously dissected. The graft consisted of the middle third of the ligament’s width, along with two bone blocks approximately 20 mm in length. After further graft preparation on the back table, femoral and tibial tunnels were created, and the graft was inserted and fixed using an interference screw (Arthrex, Naples, FL, USA) in all patients. Following graft fixation and arthroscopic verification of its position and tension, the tourniquet was released. After achieving hemostasis, the donor site was filled with PRF in the experimental group, while in Group II, the donor site was left unfilled (Figure 4). In both groups, careful reconstruction of the paratenon was performed. In the experimental group, PRF was applied to the reconstruction site and subcutaneous tissue, considering the subcutaneous localization of the infrapatellar branch. In Group II, no additional PRF instillation was performed. Subsequently, in both groups, the subcutaneous tissue and skin were sutured in the standard manner.

### 2.3. Postoperative Rehabilitation

Postoperatively, patients in both groups followed an identical rehabilitation protocol, conducted by the same team of experienced physiotherapists. Rehabilitation began on the first postoperative day and was monitored daily by a physiotherapist, continuing for four weeks post-surgery. The application of physical medicine agents led to a reduction in knee swelling, starting on the first postoperative day. Patients began with isometric and isotonic quadriceps exercises, heel props, and straight leg raises in all planes. The physiotherapist performed exercises for patellar mobilization to prevent adhesions, along with ankle pumps and retrograde massages to aid in reducing swelling. Between exercises, the leg was elevated, and ice packs were applied to the knee. Patients were able to walk using crutches with partial weight bearing on the operated leg, restricted to 30% of total body weight. All patients participating in the study adhered to this protocol. The goal for the first 15 days was to achieve a range of motion from 0° to 90°, progressing to 0° to 120° by the end of the first month. By the end of the first postoperative month, patients had regained the ability to walk independently. Progression to further stages of rehabilitation included full knee extension, straight leg raises without lag, reduction in initial joint effusion, and controlled pain. During the second month, a full range of motion was achieved, with an emphasis on strengthening the thigh muscles through water-based exercises and cycling. In the third month, exercises for strengthening the thigh muscles continued, accompanied by a transition from closed to open kinetic chain exercises. Straight-line running was introduced in the fourth month. From the sixth postoperative month onward, patients were permitted to resume running with directional changes, gradually returning to regular sports activities.

### 2.4. Postoperative Follow-Up

**Figure 5:**
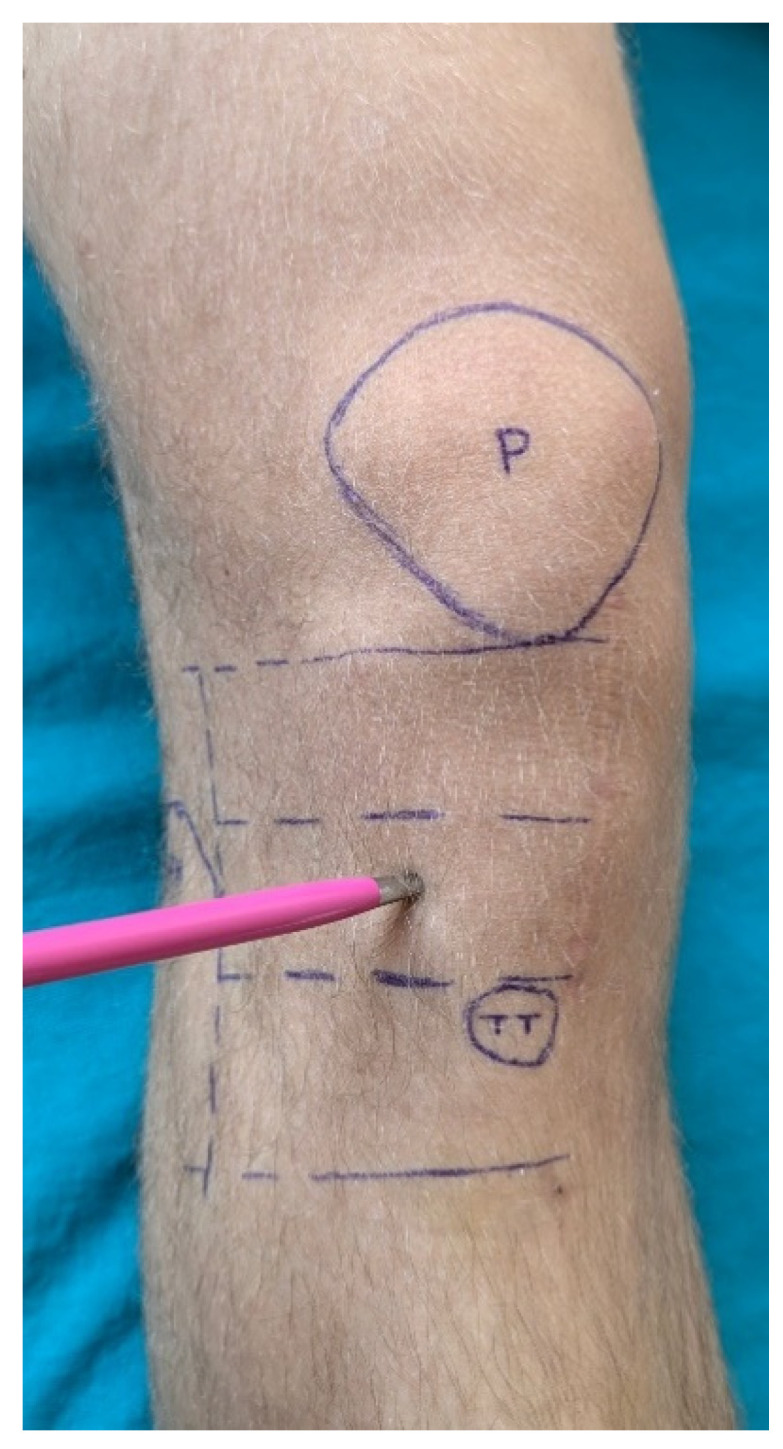
Skin region of interest for testing. P = patella; TT = tibial tuberosity.

Postoperative follow-up was conducted at four, eight and twelve months after surgery, during which clinical examinations were performed in order to assess sensory deficits. A marked area of skin on the operated knee, lateral to the surgical scar, was designated for sensory testing. The upper border of this area was at the patellar apex, the lower border was 3 cm distal to the tibial tuberosity, and the lateral border was at the anterior edge of the fibular head. This area was then divided into three horizontal sections for sensory testing (Figure 5). Sensory deficit assessment was conducted with the patient in a supine position and eyes closed during testing. The tip of a pen was used to touch three points within each horizontal section of the marked area. Patients were asked to report whether they could perceive the touch. Responses such as “I clearly feel the touch” were recorded as 0 on the test sheet if the same quality of sensation was reported at all nine points. If more than three points elicited responses such as “I don’t feel the touch” or “The touch feels altered”, the result was recorded as 1. In cases of uncertainty regarding touch perception, the same points on the contralateral knee were used for comparison. Data from the sensory testing sheets were entered into a database for statistical analysis. At the final follow-up (12 months postoperatively), patients completed subjective functional recovery assessment tests. We did not conduct quantitative sensory testing, such as electromyography (EMG) and nerve conduction studies (NCS), in this study due to practical and clinical considerations. Our facility lacks the necessary equipment and trained personnel to carry out EMG/NCS. Furthermore, we prioritized subjective testing because it is more accessible, cost-effective, and provides sufficient information for guiding clinical management in this case.

### 2.5. Statistical Analysis

The Kolmogorov–Smirnov and Shapiro–Wilk tests were used to evaluate normal distribution of data. Data were summarized using descriptive statistical methods (frequencies, percentages, mean, median, standard deviation (SD) and range). The statistical significance level was set at *p* < 0.05, with Bonferroni correction used for multiple testing within the same dataset. The Wilcoxon Rank Sum and Pearson Chi-Squared tests were used to compare characteristics between study groups. The Wilcoxon Signed-Rank, Cochrane’s Q and McNemar’s χ2 tests were used to analyze differences between measurements, both overall and within groups. The achieved power for detecting the observed difference in proportions at 12 months (PRF 0.79 vs. Standard 0.34; n = 24 vs. n = 29; two-sided α = 0.05, pooled *z*-test) was 0.93. Statistical analysis was done with the program R (version 4.3.1 (16 June 2023 ucrt)—“Beagle Scouts”; Copyright (C) 2023 The R Foundation for Statistical Computing; Platform: x86_64-w64-mingw32/x64 (64-bit)) (available at: www.r-project.org; downloaded: 21 August 2023).

## 3. Results

In the study we conducted, none of the patients presented with complications such as infection, surgical wound issues, thromboembolic events, or restrictions in the range of motion compared to contralateral side. Furthermore, clinical evaluation of the knee stability was performed at each follow-up visit, and no patient experienced graft rupture as a complication. Statistical analysis demonstrated consistency between study groups in terms of demographic characteristics, including age, body mass index (BMI), time elapsed from injury to surgery, and length of hospitalization (*p* > 0.05) (Table 1).

Functional recovery of the knee was evaluated using international scoring systems (Modified Cincinnati score, IKDC, Tegner–Lysholm score) both immediately preoperatively and at the final follow-up. The results showed a statistically significant difference in test scores between both groups (Table 2). However, in contrast to the previously mentioned tests, no statistically significant difference was observed in the values of the Tegner Activity Score when comparing the initial measurement to the final follow-up (Table 2).

Sensory deficit assessments were conducted at each follow-up visit (four, eight, and twelve months postoperatively). The results did not reveal any statistically significant differences between the groups at the first follow-up visit (*p* > 0.05). However, statistically significant differences were observed at the eight- and twelve-month follow-ups. (Table 3).

When analyzing the frequency of patients with sensory deficits across all follow-up visits, a statistically significant reduction in the incidence of sensory deficits was observed in Group 1 (defect filled with Vivostat^®^), whereas this trend was not observed in Group 2. To more precisely determine the postoperative period in which significant sensory recovery occurred, pairwise comparisons of sensory deficit incidence were performed between follow-up visits. The results indicate that significant sensory recovery in the Vivostat group occurs between eight and twelve months postoperatively, relative to the initial measurements at the four-month follow-up. This trend was not observed in the Standard group (Table 4). Based on these findings, it can be concluded that the most pronounced recovery in the Vivostat group takes place by the eighth month postoperatively, as no significant statistical differences were found between the eight- and twelve-month follow-ups postoperatively.

**Figure 6:**
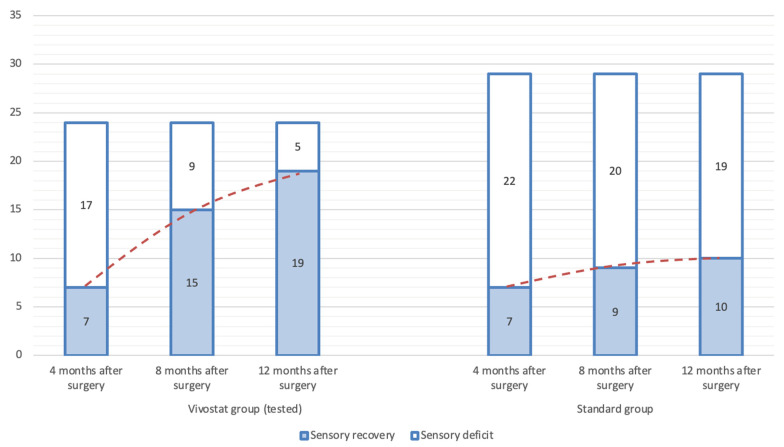
Recovery of sensory deficiency over time among groups.

The increase in patients without sensory deficits follows a progressive trend across follow-up visits. In Group 1, this trend is significantly more pronounced compared to Group 2 (Figure 6).

## 4. Discussion

This study, conducted at the XXBLINDEDXX, included a total of 53 male patients. All patients were randomized alternately into two groups, which were comparable in terms of age, body mass index (BMI), time elapsed from injury to surgery, length of hospital stay, and pre-injury level of sports activity. Upon enrollment, each patient completed a set of questionnaires assessing knee joint function four weeks prior to surgery. None of the questionnaires revealed statistically significant differences between the groups, confirming the coherence of the randomized groups.

In our study, postoperative functional recovery of the knee was assessed using international functional questionnaires (Modified Cincinnati score, IKDC, Tegner–Lysholm score), and all scores showed improvement compared to baseline values. However, no statistically significant differences were observed between the two groups, suggesting that Vivostat^®^ application does not enhance functional recovery compared to patients whose defects remained unfilled. These findings align with expectations, as sensory deficits do not impair motor abilities and therefore do not affect overall joint function.

In contrast to the demographic characteristics and functional tests, which were relatively consistent between groups, the sensory deficit assessment showed significant differences. The initial sensory deficit evaluation was conducted four months postoperatively. Statistical analysis revealed no significant differences between groups, further supporting their comparability. However, among the 17 patients in Group 1 who exhibited sensory deficits at four months postoperatively, eight demonstrated neurological improvement by the eight-month follow-up and were subsequently classified as patients without sensory deficits. Conversely, in the control group, only two of the initial 22 patients with sensory deficits showed neurological recovery at eight months postoperatively. Statistical analysis confirmed a significant difference between the groups at eight months postoperatively (*p* < 0.05). The final follow-up examination at 12 months post-surgery revealed a highly significant difference in neurological recovery, favoring Group 1 (*p* < 0.01). Of the remaining nine patients with sensory deficits, four in Group 1 experienced neurological recovery, whereas only one patient in Group 2 showed improvement. One year after surgery, 19 patients in Group 2 still exhibited sensory deficits, representing 65.5% of the total patients in that group. McNemar’s χ^2^ test was applied to evaluate neurological recovery within Group 1 across follow-ups, revealing statistically significant differences between the four-month follow-up and the eight- and twelve-month assessments. However, no significant difference was found between the eight- and twelve-month follow-ups, suggesting that Vivostat^®^ contributes to significant neurological recovery within the first eight months after surgery, after which its effect may be negligible.

Although numerous studies have explored donor site pathology following ACL graft harvesting, relatively few have focused specifically on sensory deficits. Methodological differences among studies (our and other studies) limit comparisons, as only a small number of studies with similar designs have been conducted.

In their study, Cohen et al. reported sensory deficits in surgically treated patients using the same type of graft as in our study. At six weeks postoperatively, sensory deficits were confirmed in 150 out of 218 patients included in the study, representing 69.8% of the study population [24]. This is comparable to our findings (sensory deficits were confirmed in 38 patients, accounting for 73.6% of the total sample). However, a notable difference is that Cohen et al. included all patients with sensory deficits, regardless of the graft type used for ACL reconstruction. A study conducted by Hoshi et al. included 74 patients who underwent ACL reconstruction with a BPTB graft and were followed for 12 months. However, the reported incidence of hypesthesia and anesthesia was significantly higher compared to our study—93%. Similarly, Barie et al. reported significantly higher donor site morbidity following the use of a BPTB graft compared to a quadriceps tendon graft, particularly in terms of kneeling pain, with no clear data on sensory deficits [25]. The authors of the study acknowledged that the reported pain could be related to nerve injury, though they did not consider nerve preservation to be clinically relevant, nor did they investigate nerve recovery further. A conceptually similar study utilizing bioregenerative medicine products was conducted by Cervelin et al., who filled the donor site following BPTB graft harvesting with PRP, aiming to reduce donor site morbidity. They assessed morbidity by addressing both the bony and ligamentous donor sites and interpreting the outcomes through pain reduction [26]. Although methodologically similar, the study did not consider sensory deficit as part of donor site morbidity and therefore cannot be directly compared to our study. Kovindha et al. specifically investigated sensory deficits by measuring the affected skin area in 35 patients. They concluded that between 3 and 6 months postoperatively, the area of sensory deficit decreased from an average of 12.8 to 3.3 cm^2^ (*p* < 0.01), with the defect filled using an autologous bone graft [27]. Unfortunately, our study is not directly comparable to any existing studies involving the filling of bone-tendon defects using either grafts or bioregenerative products, as previously published research has not focused on sensory deficits, instead emphasizing radiographic recovery and the impact of these products on prepatellar pain.

In addition to the use of bioregenerative medicine products, a study conducted by Tsuda et al. demonstrated that using two horizontal incisions during graft harvesting can reduce the incidence of infrapatellar branch injury. As a result, symptoms were observed in only 17% of the 93 patients included in the study, with bone defects also being grafted following the reconstruction [28]. Mishra et al. also discussed the use of two horizontal incisions as a method for nerve protection, with an acceptable cosmetic outcome. However, the study itself provides no data regarding nerve function [29]. Additionally, studies conducted by Kartus et al. and Gaudot et al. demonstrated a statistically significant reduction in the area of sensory deficit following the use of two horizontal incisions compared to a single vertical incision for graft harvesting [10,30]. Kartus et al. used a transtibial approach, while Gaudot et al. employed an anatomical reconstruction technique. Both studies measured sensory deficits in square centimeters and focused on minimizing the risk of nerve injury, making direct comparison with our study difficult. Reducing the incision length along with meticulous soft tissue dissection can help minimize the risk of nerve injury during surgical graft harvesting.

There are several limitations to our study. The most evident are the relatively small sample size and the short follow-up period of only one year, which could be mitigated by additional follow-ups and the inclusion of more patients in future studies. By including only male patients, we also leave open the possibility of different outcomes in female patients. Another limitation of this pilot study is that the beneficial effect of PRF on sensory recovery appeared to plateau after eight months, which should be carefully considered when interpreting long-term outcomes. Furthermore, since the assessment is based on the patient’s subjective perception, we encourage performing quantitative sensory testing and electrophysiological confirmation, as its use would further improve the strength of the study.

## 5. Conclusions

Our study may be the first to directly address iatrogenic injury of the infrapatellar branch of the saphenous nerve, focusing exclusively on sensory deficit in the context of donor site morbidity, following the use of a BPTB graft in ACL reconstruction. Most previous studies have primarily focused on anterior knee pain, particularly pain occurring during kneeling. While this does not impact the functional outcome of treatment, it compromises the patient’s quality of life and should not be disregarded. The results of our study are encouraging, as they demonstrate a reduction in the number of patients experiencing symptoms, thereby improving overall treatment outcomes and patient satisfaction. Additionally, the use of Vivostat^®^ is simple and does not significantly extend the duration of surgery. Despite the spontaneous resolution of symptoms in some patients, modern medicine enables faster relief and greater effectiveness across a larger patient population.

Although it is nearly impossible to completely avoid injury to the infrapatellar branch due to individual variability in the branching of the saphenous nerve, this risk can be reduced, and the consequences can be actively managed if they do occur. Given our belief in the potential of bioregenerative medicine, we strongly encourage further investigation of PRF in long-term follow-up studies, as well as the use of other bioregenerative products in future research.

## Figures and Tables

**Table 1:** Patients’ characteristics.

Characteristics	Total	Vivostat	Standard	Test
Gender				
Male	53 (100%)	24 (100%)	29 (100%)	
Age (years)				ns *
Mean (SD)	27.13 (8.47)	26.54 (9.55)	27.62 (7.6)	
Median (Range)	25 (17–52)	24 (17–52)	26 (17–44)	
BMI (kg/m^2^)				ns *
Mean (SD)	25.68 (2.56)	25.48 (2.43)	25.84 (2.7)	
Median (Range)	24.97 (21.61–35.35)	24.88 (21.61–31.24)	24.97 (22.92–35.35)	
Length of time ^#^ (months)				ns *
Mean (SD)	13.21 (22.01)	16.97 (30.32)	10.09 (11.06)	
Median (Range)	7 (0.07–144)	7 (0.36–144)	8 (0.07–42)	
Length of hospitalization				ns *
Mean (SD)	5.19 (3.06)	4.92 (2.19)	5.41 (3.66)	
Median (Range)	4 (2–20)	4 (3–11)	4 (2–20)	
**Total**	53 (**100%**)	24 (100%)	29 (100%)	

ns—not statistically significant; ^#^ Length of time between injury and treatment; * Wilcoxon rank-sum test.

**Table 2:** Preoperative and postoperative results of the subjective tests.

Type of Score	Total	Vivostat	Standard	Test *
**Modified Cincinnati score**				
Initial (preoperative) values				
Mean (SD)	64.4 (21.66)	69.5 (17.91)	60.17 (23.81)	ns
Median (Range)	65 (12–100)	74 (34–100)	62 (12–91)	
12 months after surgery				
Mean (SD)	93.3 (7.45)	92.67 (7.91)	93.82 (7.15)	ns
Median (Range)	96 (70–100)	94.5 (75–100)	96 (70–100)	
Wilcoxon Signed-Rank Test				-
12 months vs. initial	***p* < 0.01**	***p* < 0.01**	***p* < 0.01**	
**IKDC score**				
Initial (preoperative) values				
Mean (SD)	57.4 (19.25)	60.62 (17.16)	54.74 (20.73)	ns
Median (Range)	59.8 (14.9–93.1)	61.5 (21.8–88.5)	56.3 (14.9–93.1)	
12 months after surgery				
Mean (SD)	89.72 (9.44)	90.33 (8.86)	90.33 (8.86)	ns
Median (Range)	89.7 (52.9–100)	89.7 (67.8–100)	89.7 (67.8–100)	
Wilcoxon Signed-Rank Test				-
12 months vs. initial	***p* < 0.01**	***p* < 0.01**	***p* < 0.01**	
**Tegner–Lysholm Score**				
Initial (preoperative) values				
Mean (SD)	71.83 (20.36)	75.75 (17.06)	68.59 (22.51)	ns
Median (Range)	78 (16–100)	80.5 (39–100)	77 (16–94)	
12 months after surgery				
Mean (SD)	94.25 (12.7)	95.75 (5.2)	93 (16.54)	ns
Median (Range)	95 (10–100)	97 (81–100)	95 (10–100)	
Wilcoxon Signed-Rank Test				-
12 months vs. initial	***p* < 0.01**	***p* < 0.01**	***p* < 0.01**	
**Activity score**				
Initial (preoperative) values				
Mean (SD)	6.45 (2.64)	6.67 (2.7)	6.28 (2.62)	ns
Median (Range)	7 (0–10)	7 (2–10)	7 (0–10)	
12 months after surgery				
Mean (SD)	6.26 (2.1)	6.5 (2.02)	6.07 (2.17)	ns
Median (Range)	6 (1–10)	6 (3–10)	6 (1–10)	
Wilcoxon Signed-Rank Test				-
12 months vs. initial	ns	ns	ns	

ns—not statistically significant; * Wilcoxon rank-sum test.

**Table 3:** Sensory deficits between groups.

Sensory Deficit	Total	Vivostat	Standard	Test *
4 months after surgery				
Sensory 0	14 (26.42%)	7 (29.17%)	7 (24.14%)	ns
Sensory 1	39 (73.58%)	17 (70.83%)	22 (75.86%)	
8 months after surgery				
Sensory 0	24 (45.28%)	15 (62.5%)	9 (31.03%)	***p*** ** < 0.05**
Sensory 1	29 (54.72%)	9 (37.5%)	20 (68.97%)	
12 months after surgery				
Sensory 0	29 (54.72%)	19 (79.17%)	10 (34.48%)	***p*** ** < 0.01**
Sensory 1	24 (45.28%)	5 (20.83%)	19 (65.52%)	
**Total**	53 (100%)	24 (100%)	29 (100%)	-

ns—not statistically significant; * Pearson χ^2^ tests.

**Table 4:** Sensory deficit between controls.

Sensitivity (Control Exams)	McNemar’s χ^2^ Test		
	Total	Vivostat	Standard
4 m vs. 8 m vs. 12 m	*p* ^#^ = 8.6 × 10^−6^	*p* ^#^ = 8.8 × 10^−5^	ns ^#^
4 m vs. 8 m	*p * * = 0.0044	*p * * = 0.0133	*p* * = 0.4795
4 m vs. 12 m	*p * * = 0.0003	*p * * = 0.0015	*p* * = 0.2482
8 m vs. 12 m	*p* * = 0.0736	*p* * = 0.1336	*p* * = 1

* The Bonferroni correction: 0.05/3 = 0.0167; ns—not statistically significant; m—months; ^#^ Cochrane’s Q test.

## Data Availability

All data used in this study can be obtained via a request to the following email: darkomil@doctor.com.

## Abbreviations

The following abbreviations are used in this manuscript:

BPTB	Bone–patellar tendon–bone
ACL	Anterior cruciate ligament
IPBSN	Infrapatellar branch of the saphenous nerve
PRF	Linear dichroism
PRP	Platelet-rich fibrin
IKDC	International Knee Documentation Committee
SD	Standard deviation
BMI	Body mass index
